# Building consensus on MIGS: insights from a UKEGS survey

**DOI:** 10.1038/s41433-025-03852-9

**Published:** 2025-05-20

**Authors:** Abdus Samad Ansari, Andrew J. Tatham, Nishani Amerasinghe, Gus Gazzard

**Affiliations:** 1 Moorfields Eye Hospital, NHS Foundation Trust, London, UK; 2 University of Edinburgh, Edinburgh, UK; 3 Princess Alexandra Eye Pavilion, 45 Chalmers Street, Edinburgh, UK; 4 University Hospital Southampton, Hampshire, UK; 5 Institute of Ophthalmology, University College London, UCL, London, UK; 6 NIHR Moorfields Biomedical Research Centre, 162 City Road, London, UK

**Keywords:** Optic nerve diseases, Physiology, Education

## Introduction

Minimally invasive glaucoma surgery (MIGS) offers safer, less invasive alternatives to traditional surgical procedures [1]. Advantages include faster recovery, shorter operations and reduced medication burden [2] via trabecular-bypass or suprachoroidal routes [3]. There is increasing evidence of efficacy justifying adoption and integration into modern glaucoma care [4], yet considerable inconsistency in practice remains, emphasising the need for guidelines [5].

## Methods

Members of UK and Eire Glaucoma Society (UKEGS) completed a nationwide survey (see supplementary material) to assess variations in practice and gaps in current policies on MIGS. The findings provide valuable insights to inform consensus-driven recommendations, guide professional training, and support the development of standardised approaches to MIGS and patient care.

## Demographics

A nationwide survey of all glaucoma specialist members of UKEGS at the time of distribution in 2024 received 80 responses, representing 20% of The Royal College of Ophthalmologists (RCOphth) members that have specified their primary SIA in the workforce census 2022. These surgeons reported performing 36,600 cataract procedures annually, a mean of 458 per surgeon, with a combined surgical experience of 1,338 years (mean:17). Most (54%, *n *= 43) were in tertiary care centres, 38% (*n *= 30) were in district general hospitals and 6% (*n *= 5) in purely private institutions. Respondents performed on average 75 conventional glaucoma surgeries annually (trabeculectomies, microshunt and aqueous shunt implantations). A complete background of respondents can be seen in Table 1.

**Table 1:** Respondent’s background.

Question:			Total Responses
Role	*Consultant*: 78.48% (62)		79
	*Trainee*: 7.59% (6)		
	*Other*: 13.92% (11)		
Hospital Setting	*Tertiary*: 53.75% (43)		80
	*District*: 37.50% (30)		
	*Private*: 6.25% (5)		
	*Other*: 2.50% (2)		

	Average	Total	
Cataract Procedures completed each year	458	36,600	80
Number of conventional glaucoma drainage procedures per year?\	75	6030	80
Number of years performing cataract surgery on average	17	1338	80
	**Yes**	**No**	
Carry out cataract surgery in glaucoma patients	97.50% (78)	2.50% (2)	80
Use MIGS in current practice	89.74% (70)	10.26% (8)	78
Use ‘stand-alone’ MIGS procedures	57.50% (46)	42.50% (34)	80

## Key insights and trends

There was a firm belief that MIGS procedures have an important role in glaucoma management and that they can slow vision loss (95%, *n *= 76), reduce the need for further pressure-lowering incisional glaucoma surgery (94%, *n *= 75) and lower the burden of medical therapy (98%, *n *= 78). There were some differences in opinion about whether MIGS might divert resources from more critical areas, with 57% (*n *= 45) expressing this concern, whilst 44% (*n *= 35) disagreed: highlighting the need for robust cost-effectiveness data to guide resource allocation.

Despite the integration of MIGS with cataract surgery becoming increasingly routine, there remains wide variation in patient selection. When asked who should be offered MIGS procedures, responses varied: 33% (*n *= 26) favoured offering MIGS to a minority of carefully selected patients, 55% (*n *= 44) supported use for all patients taking intraocular pressure-lowering medications, 16% (*n *= 13) advocated offering MIGS to *all* glaucoma patients and 5% (*n *= 4) selected none of these options. These findings emphasise the need to establish clear guidelines for standardising patient selection, ensuring that the treating surgeon possesses a comprehensive understanding of glaucoma progression, risk assessment, and alternative treatments options. Such expertise is typically limited to those trained in the subspecialty. When asked whether MIGS procedures should be confined to glaucoma specialists, 88% (*n *= 70) agreed, 85% (*n *= 67) believed MIGS should not be carried out by surgeons whose primary focus is cataract surgery. This is a valid concern: while carrying out MIGS may be technically feasible, selecting the correct procedure is more complex. The growing range of devices and techniques—often lacking RCT evidence—means balancing immediate risks against long-term benefits demands a detailed understanding of prognosis as well as surgical expertise.

Our results reveal strong support from glaucoma specialists for standardising practice through national guidelines and prioritising patient transparency. A significant majority (86%, *n *= 68) believe MIGS should be subject to national guidelines informed by clinical RCTs and 85% (*n *= 67) that cost-effectiveness be incorporated. Regarding patient communication, 96% (*n *= 77) of respondents believe that glaucoma patients have the right to be informed about the availability of MIGS on the NHS, particularly when these devices are CE-marked, FDA-approved and supported by robust evidence from RCTs, Cochrane reviews and NICE guidance. Additionally, 84% (*n *= 67) believe surgeons have a duty to inform patients if they are undergoing a novel procedure without supportive RCT evidence, while 83% (*n *= 66) agreed patients should also be informed if a technique is new to the surgeon.

## Areas of concern

Our findings raise concerns about independent sector treatment centres (ISTCs) performing MIGS or cataract surgery in glaucoma patients. Though vital for reducing backlogs and improving access, ISTCs focus on high-volume, low-complexity cases under a fee-for-service model. This suggests ISTCs may not be best placed to provide the specialised expertise for the complexities of selecting appropriate MIGS and still less so the careful provision of long-term follow-up patients require. This survey identifies significant concerns about non-specialists managing high-risk glaucoma patients. To mitigate these risks, stricter oversight and restriction of MIGS to trained specialists is strongly advocated. Forthcoming Royal College of Ophthalmology guidelines on MIGS procedures are to be welcomed.

A major concern is the lack of counselling about MIGS during surgical consultations for glaucoma patients. Not discussing these procedures risks missed opportunities to optimise intraocular pressure control, reduce medication burden, and enhance quality of life. Standardising this discussion could help reduce disparities in access and ensure equitable, comprehensive care.

## Future outlook

Our results indicate a generally optimistic outlook regarding MIGS’s adoption and future utilisation. Most respondents (61%, *n *= 48) deemed the process of introducing these new procedures achievable, with 30% (*n *= 23) describing it as straightforward. However, 10% (*n *= 8) identified the process as challenging, reflecting mixed institutional readiness. Looking ahead, 78% (*n *= 62) anticipated an increase in the use of MIGS at their respective hospitals, signalling growing confidence in its clinical benefits and integration into glaucoma management.

## Conclusion

MIGS has been widely adopted by glaucoma specialists, with strong support for its benefits in treating the disease. The consensus around combining MIGS with cataract surgery indicates its established efficacy and safety. However, there is inequality of access, with variability in practice and implementation, potential gaps in training, patient education, or institutional policies. Based on these findings, the UKEGS recommends the following guidelines:

- The decision to perform MIGS should be made by the clinician overseeing a patient’s long-term glaucoma care, with the procedure only being performed by surgeons with specialist training and experience in managing the condition over time
- Ensure all glaucoma patients undergoing cataract surgery are offered MIGS and made aware of the potential benefits.
- Develop standardised materials to educate patients on MIGS and the evidence to help decision-making.
- Limit MIGS use in independent sector treatment centres (ISTCs) to surgeons with glaucoma fellowship training.

## Supplementary information

Supplemental Material

## Data Availability

All data generated or analysed during this study are included in this published article
