# Supplementary material for: Building consensus on MIGS: insights from a UKEGS survey
Source: Eye (Lond). 2025 May 20;39(11):2107–9. doi: 10.1038/s41433-025-03852-9 (PMC12274436; doi:10.1038/s41433-025-03852-9)
Supplement: Supplementary file 1 — Supplemental Material [file 41433_2025_3852_MOESM1_ESM.docx]

# UKEGS MIGS Survey Questions with Response Types and Options

The 17 primary questions from the UKEGS MIGS usage survey, including sub-questions and response types. Where applicable, the complete set of multiple-choice response options is also provided.

| **Question #** | **Question** | **Response Type (Options)** |
| --- | --- | --- |
| **Q1** | Do you carry out cataract surgery in glaucoma patients? | Yes/No |
| **Q2** | Do you use MIGS? | Yes/No |
| **Q3** | Do you use 'stand-alone' MIGS? | Yes/No |
| **Q4** | Do you use MIGS in combination with cataract surgery? | Yes/No |
| **Q5a** | Do you believe that MIGS can slow the rate of vision loss? | Yes/No |
| **Q5b** | Do you believe that MIGS can reduce the need for further pressure-lowering incisional glaucoma surgery? | Yes/No |
| **Q5c** | Do you believe that MIGS can lower the burden associated with topical medical therapy? | Yes/No |
| **Q5d** | Do you believe that MIGS can divert valuable resources from more important areas? | Yes/No |
| **Q6** | Which glaucoma patients (if any) do you consider should currently be offered MIGS surgery at the time of cataract surgery? | *Multiple Choice:* - None of the above - No-one - Only a minority group of selected patients - All glaucoma patients currently requiring IOP lowering medications - All glaucoma patients |
| **Q7a** | Should access to MIGS be restricted to glaucoma specialists only? | Yes/No |
| **Q7b** | Should MIGS be available for use by cataract surgeons? | Yes/No |
| **Q7c** | Should MIGS use be subject to national guidelines guided by clinical RCT data? | Yes/No |
| **Q7d** | Should MIGS use be subject to national guidelines guided by RCTs and cost-effectiveness data? | Yes/No |
| **Q8** | Do you believe that glaucoma patients should be informed that MIGS* are available on the NHS? (*CE marked, FDA approved, with supporting evidence from randomised controlled trials, COCHRANE Library reviews and NICE Guidance) | Yes/No |
| **Q9** | Do you think that surgeons have a duty to inform patients undergoing a novel technology without supportive RCT evidence that this is the case? | Yes/No |
| **Q10** | Do you think that surgeons have a duty to inform patients undergoing a technique which is new to the surgeon that this is the case? | Yes/No |
| **Q11** | What kind of hospital do you work in? | *Multiple Choice:* - District General Hospital - Tertiary Referral Hospital - Private Hospital - Other |
| **Q12** | Approximately, how many cataract procedures do you perform each year? | Numerical (Average/Total) |
| **Q13** | Approximately, how many conventional drainage glaucoma procedures (Trab/Tube/Preserflo/XEN etc) do you perform each year? | Numerical (Average/Total) |
| **Q14** | What is your role? | Multiple Choice: - Consultant - Trainee - Other |
| **Q15** | How many years have you been performing Cataract surgery? | Numerical (Years) |
| **Q16** | How would you describe the process for introducing new procedures (such as MIGS) into your hospital? | *Multiple Choice:* - Straightforward - Difficult but achievable - Difficult to the extent that I am not motivated to try |
| **Q17** | Based on the future outlook for your hospital, do you anticipate that usage of MIGS: | *Multiple Choice:* - Increase - Stay the same - Reduce |
